# Chediak-Higashi Syndrome: A Case Report of a Girl Without Silvery Hair and Oculocutaneous Albinism Presenting with Hemophagocytic Lymphohistiocytosis

**DOI:** 10.4274/tjh.2014.0049

**Published:** 2014-12-05

**Authors:** Murat Elevli, Halil Uğur Hatipoğlu, Mahmut Civilibal, Nilgün Selçuk Duru, Tiraje Celkan

**Affiliations:** 1 Sakarya University Faculty of Medicine, Clinic of Pediatrics, Sakarya, Turkey; 2 Haseki Education and Research Hospital, Clinic of Pediatrics, İstanbul, Turkey; 3 İstanbul University Cerrahpaşa Faculty of Medicine, Department of Pediatric Hematology-Oncology, İstanbul, Turkey

**Keywords:** Chediak-Higashi syndrome, Hemophagocytic lymphohistiocytosis, Oculocutaneous albinism, Lyst gene, immune deficiency

## TO THE EDITOR

Chediak-Higashi syndrome (CHS) is a rare, autosomal recessive inherited disorder characterized by variable degrees of oculocutaneous albinism, severe immune deficiency and unassociated lymphoproliferative syndrome, and intracytoplasmic giant granules in leukocytes, monocytes, platelets, melanocytes, and erythroid precursors [1,2,3,4,5]. CHS is caused by mutations in the lysosomal trafficking regulator gene (LYST) [3,6]. The role of the LYST gene in the trafficking of granules results in defective release of melanin or cytolytic enzymes, causing hypopigmentation of the skin and hair as well as cytotoxic defect [3]. There are 2 clinical periods of the disease: stable and accelerated. In the accelerated phase, fever, hepatosplenomegaly, hepatitis, lymphohistiocytic infiltration, pancytopenia, coagulopathy, hemorrhage, and peripheral neuropathy are seen [1]. Herein, we report a case of CHS presented with hemophagocytic lymphohistiocytosis (HLH).

A 5-month-old girl presented with fever. She was febrile and pale, and she had splenomegaly with no hepatomegaly. There were no neurological symptoms, lymphadenopathy, or bleeding signs. Her body temperature was over 38.8 °C for 8 days in spite of ampicillin/sulbactam and netilmicin therapy. Hematological investigation revealed hemoglobin of 6.3 g/dL, white blood cell count of 3.8x109/L (70% lymphocytes, 10% neutrophils), and platelet count of 84x109/L. Erythrocyte sedimentation rate was 25 mm/h, C reactive protein was 6.75 mg/dL, and ferritin was 635 ng/mL, with a normal fibrinogen level. Serum triglyceride level was 584 mg/dL with a normal serum cholesterol level. Serum lactate dehydrogenase, serum glutamic oxaloacetic transaminase, and serum glutamic pyruvate transaminase levels were 882 U/L, 254 U/L, and 54 U/L, respectively. Her fever was controlled with piperacillin/tazobactam and amikacin combined with fluconazole. Serological markers were negative for cytomegalovirus, Epstein-Barr virus, human immunodeficiency virus, parvovirus, Toxoplasma, rubella, and hepatitis B virus. Salmonella and Brucella tube agglutination test results were also negative. Blood and urine cultures did not demonstrate any infectious agents. Tandem MS, blood amino acid levels, urinary organic acid profile, and urinary mucopolysaccharide screening tests were all normal. On bone marrow aspiration smear, hemophagocytosis was observed. The patient was diagnosed with HLH and the HLH-2004 treatment protocol, including dexamethasone and etoposide, was administrated. No gene defect was determined on exons 2 and 3 of the perforin 1 coding region. On both bone marrow aspiration and peripheral blood smears, intracytoplasmic giant granules in lymphocytes and monocytes were observed. Hair analysis for CHS was consistent with CHS. At the end of the initial therapy, the disease was in remission. Continuation therapy of the HLH regimen was then started. Stem cell transplantation (SCT) has been planned. As there was no matched related donor for SCT at that time, we will be treating her according to HLH-2004 continuation therapy until a matched or mismatched unrelated donor is found. Informed consent was obtained.

**Figure 1:**
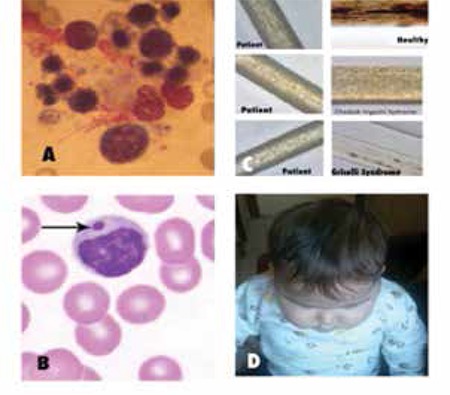
A. Hemophagocytosis on bone marrow smear.B. An intracytoplasmic giant granule in a monocyte on peripheral blood smear.C. Light microscopic image of the hair shaft shows abnormal clumping of melanin in patient’s hair. Pigment clumbs are small and uniformly and regularly distributed along the hair shaft. This is consistant with Chediak-Higashi syndrome.D. Our patient does not have oculocutaneous albinism and she is black-haired.

Our patient was diagnosed with HLH because there was hemophagocytosis on bone marrow aspiration (Figure 1a) and she had splenomegaly, fever, pancytopenia, hypertriglyceridemia, and high ferritin levels. Sometimes the CHS diagnosis can be considered only after the observation of gray hair and giant intracellular granules on a peripheral blood smear, and light microscopy of a hair shaft can facilitate a quick diagnosis [2]. We thought that the diagnosis could be CHS because of the bone marrow aspiration and peripheral blood smears findings (Figure 1b). Light microscopic images of the patient’s hair showed abnormal clumping of melanin. Pigment clumps were small and uniformly and regularly distributed along the hair shaft (Figure 1c). Our patient was therefore diagnosed by hair analysis although she does not have the CHS phenotype (Figure 1d).

Patients with CHS may have a variable clinical presentation due to different mutations in the LYST gene. Nonsense and frameshift mutations of the LYST gene are associated with severe early-onset childhood CHS and characterized by fatal infections and HLH. Meanwhile, missense mutations of the same gene are associated with milder, late-onset CHS with slowly progressive neurological impairment or an adolescent form with infections but no HLH [2,6,7]. In our case there is probably a mutation of the LYST gene that is associated with the normal phenotype and the specific findings of CHS on peripheral blood smear, bone marrow aspiration smear, and light microscopic image of the hair shaft but causes early-onset HLH. Genetic analysis is needed to explain this exactly.

In our case there was no specific treatment for CHS. Intravenous antibiotherapy, erythrocyte suspension, thrombocyte suspension, and, in accordance with the HLH 2004 protocol, dexamethasone and etoposide were used. The patient has been positively responsive to chemotherapy and, as the main treatment of CHS, SCT is planned, but a donor has not yet been found [8].

In conclusion, we think that it is not necessary to find oculocutaneous albinism and silvery hair in all patients to diagnose CHS. Bone marrow aspiration and peripheral blood smears and light microscopic images of the hair shaft may be sufficient for diagnosis in some cases without genetic analysis.

**Conflict of Interest Statement**

The authors of this paper have no conflicts of interest, including specific financial interests, relationships, and/or affiliations relevant to the subject matter or materials included.
